# Equilibrium Separation of Siloxanes in Metal–Organic Frameworks

**DOI:** 10.1021/acs.jpcc.5c04283

**Published:** 2025-09-04

**Authors:** Jia Yuan Chng, David S. Sholl

**Affiliations:** † School of Chemical & Biomolecular Engineering, 1372 Georgia Institute of Technology, Atlanta, Georgia 30332-0100, United States; ‡ 6146 Oak Ridge National Laboratory, Oak Ridge, Tennessee 37830, United States

## Abstract

We present an *in silico* assessment of metal–organic frameworks (MOFs) for the equilibrium separation of linear and cyclic siloxanes. Using a combination of configurational bias/continuous fractional component Monte Carlo (CB/CFCMC) simulations and Ideal Adsorbed Solution Theory (IAST), we investigated the adsorption of both equimolar and nonequimolar mixtures of linear and cyclic siloxanes in a selection of synthesizable MOFs with medium to large pore volumes. We showed that configurational entropy effects drive the preferential adsorption of linear siloxanes over cyclic siloxanes. Based on synthesizability metrics we identified ZIF-70 as a promising adsorbent for the separation of linear and cyclic siloxanes. Self-diffusivities of linear and cyclic siloxanes in ZIF-70 calculated from molecular dynamics simulations show that equilibrium can be reached on reasonable time scales. We explored vacuum-temperature swing adsorption (VTSA) as a potential method for the recovery of adsorbed linear siloxanes from ZIF-70, achieving a 75% reduction in adsorbed phase concentration. Additionally, we demonstrated that supercritical CO_2_ offers an alternative desorption strategy by displacing adsorbed linear siloxanes in ZIF-70 at pressures above 200 bar driven by entropy effects.

## Graphical abstract

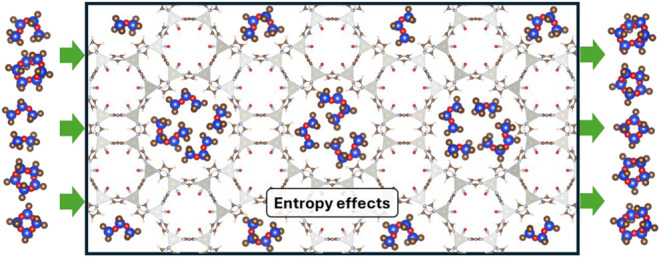

## 1 Introduction

Silicone polymers are integral in various industrial applications, including electronics^1^ and construction,^2^ biomaterials and medical devices^3,4^ due to their physicochemical stability. Cyclic siloxanes are monomers in the manufacturing of silicone polymers.^5^ The volatile nature of cyclic siloxanes prompted health and environmental investigations by regulatory agencies, environmental groups and the silicone industry. Numerous studies have focused on understanding the presence of silicones^6,7^ and their degradation mechanisms in the environment.^8,9^ Despite significant research, many questions regarding the environmental and health impacts of cyclic siloxanes remain unanswered, leading to varied regulatory responses worldwide. For example, the European Chemicals Agency has banned the use of D4 in cosmetic products due to concerns about its persistence, bioaccumulation, and potential reproductive toxicity.^10,11^ Conversely, countries such as Australia, Japan, and the United States have adopted more lenient measures, and China is still in the process of finalizing its assessment, contributing to a complex global regulatory environment.^12^ These observations motivate the need to remove residual cyclic siloxanes from silicone polymers.

Currently, fractional distillation or stripping is employed to remove residual cyclic siloxanes (e.g., D4, D5, D6) from silicone polymers.^13^ Finding less energy-intensive processes to achieve this goal would be beneficial. A recent study on membrane separations demonstrated that polymeric membranes are able to remove cyclic siloxanes from silicone polymers.^14^ During this process, short-chain linear siloxanes may also diffuse through the membrane along with cyclic siloxanes, resulting in a permeate mixture of both classes of molecules. No information is currently available on the membrane’s selectivity for linear siloxanes. We assume that the membrane separation system is able to remove D4, D5 and D6 from silicone polymers but also allows the permeation of L2, L3 and L4 siloxanes. This paper focuses on the equilibrium separation of short linear siloxanes (L2, L3, L4) from cyclic siloxanes (D4, D5, D6) using adsorption-based separations as an alternative to distillation.

Existing literature on adsorption of siloxanes in porous materials focuses on the removal of specific cyclic siloxanes, typically D4, from biogas using activated carbon as an adsorbent.^15,16^ Despite its effectiveness, activated carbon’s limited regenerability restricts its broader application. Silica gel^17,18^ and Metal–Organic Frameworks^19–21^ (MOFs) have demonstrated good regenerability for D4 adsorption at high temperatures and deep vacuum levels, respectively. Computational and experimental studies by Gulcay-Ozcan et al. showed that MOF PCN-777 outperforms DUT-4 ^19^ and MIL-101 ^20^ in both adsorption capacity and regenerability.^22^

The large number of MOFs and related materials that exist means that it is interesting to explore which of these materials are well suited for siloxane separations. Direct experimental testing of large numbers of materials is resource intensive, so computational modeling can be useful if models with sufficient accuracy are available. Our recent work introduced accurate force fields (FFs) for siloxane adsorption in MOFs and for simulations of bulk siloxane phases, showing by comparison with experimental VLE data and Density Functional Theory calculations for adsorption that earlier FFs were inaccurate.^23^ The availability of these FFs means that molecular simulations can be used to make useful predictions about the separation performance of MOFs for siloxanes. The separation of linear and cyclic siloxanes can be achieved by the preferential adsorption of either class of siloxanes. We previously employed molecular dynamics simulations to identify MOFs for the kinetic separation of cyclic and linear siloxanes by exploiting the differences in size between these two classes of siloxanes.^24^ In this paper we turn our attention to equilibrium separations, which offer an alternative approach where selectivity can be driven by adsorption affinity or entropy effects in MOFs with pores that are large enough to allow diffusion of all siloxanes of interest.

No information is currently available on the equilibrium separation of linear and cyclic siloxanes using MOFs. The potential for MOFs as adsorbents for equilibrium separation has been demonstrated with other classes of molecules. Most existing literature on computational screening of MOFs for adsorption-based separations focuses on binary gaseous mixtures such as CO_2_/N_2_,^25,26^ CO_2_/H_2_,^27,28^ CO_2_/CH_4_,^27,29^ CH_4_/H_2_,^30,31^ noble gases,^32,33^ olefin/paraffin^34,35^ and O_2_/N_2_.^36^ These separations typically operate at conditions well below pore saturation, with selectivity relying on differences in binding strengths.^37^ Studies using computational screening for multicomponent mixtures are less common. For example, several research groups have applied different strategies to discover promising MOFs for the separation of five-component hexane isomers (*n*-hexane/2-methylpentane/3-methylpentane/2,3-dimethylbutane/2,2-dimethylbutane). Chung et al. initially used Widom insertion calculations to identify MOFs and zeolites with high selectivity in the infinite-dilution limit at 433 K for this mixture, followed by GCMC simulations for equimolar mixture uptakes at 433 K and 1 bar.^38^ In an alternative approach, Solanki et al. and Peng et al. first selected MOFs with structural properties (PLD and surface area) within a prespecified range before performing GCMC simulations of equimolar hexane isomer adsorption in thousands of MOFs at 433 K and 10 bar.^39,40^ A possible weakness of these approaches is that it is unclear whether properties in the infinite dilution limit are useful predictors of adsorption of mixtures from liquid mixtures. Another possible weakness is that these approaches assumed adsorption is fully reversible without simulating a complete cyclic process. Mixture separations near pore saturation conditions are commonly driven by entropy effects,^37^ a phenomenon that explains the preferential adsorption of smaller adsorbates in some materials at high loadings. For example, dibranched hexane isomers are preferentially adsorbed over the linear and monobranched hexane isomers at high pore loadings because they are more compact, resulting in greater packing effects.^39,41,42^ This effect was also observed with the high uptake of C6–C8 *n*-alkanes compared to *n*-nonane in MIL-100­(Cr) and MIL-101­(Cr).^43^ Krishna demonstrated the application of entropy-based principle to the separation of various binary mixtures (*n*-alkanes, hexane isomers, aromatics, *n*-alcohols) in zeolites and MOFs.^37^

In our previous work, we demonstrated using molecular simulations that an entropy effect drives the separation of binary linear and cyclic siloxane mixtures in a representative large-pore MOF, FOTNIN.^23^ In this material, linear siloxanes were preferentially adsorbed due to their ability to pack more efficiently in the MOF’s pores than cyclic siloxanes. In this work, we use a combination of computational simulation techniques to study the coadsorption of more complex multicomponent mixture of linear and cyclic siloxanes in MOFs with pores that will allow passage of both classes of siloxanes. Unlike previous studies that focused on binary mixtures, this work examines the separation of a six-component mixture of linear and cyclic siloxanes, providing a more comprehensive understanding of the adsorption among siloxanes with similar and different molecular weights and shapes. While the preferential adsorption of linear siloxanes in FOTNIN demonstrated entropy effects in separation, the practical application of FOTNIN, a Zr–porphyrin MOF, may be difficult because of challenges in reproducibility.^44^ Therefore, this work aims not only to confirm the separation mechanism in a more complex six-component mixture but also to identify new and practical MOFs that can efficiently accomplish the separation of linear and cyclic siloxanes.

## 2 Methods

### 2.1 MOF Structures

MOF structures were sourced from the QMOF database.^45^ The Zeo++ software^46^ was used with high precision settings to compute the helium accessible void fraction and identify any open metal sites in the MOFs. From a large pool of structures in the QMOF database, we first excluded MOFs with open metal sites, since they might interact strongly with polar molecules^47^ such as carbon dioxide^48,49^ and water vapor^50^ that may be present in siloxane mixtures. To ensure that the selected MOFs would allow the diffusion of both linear and cyclic siloxanes, we considered only materials with pore limiting diameters (PLD) larger than 9 Å. This criterion is based on findings from our previous work, where we observed that L2, L3, L4, D4, D5 and D6 siloxanes could diffuse through rigid 1D MOFs with a PLD of 7.7 Å or larger.^24^ By only considering MOFs with PLD greater 9 Å, we aim to ensure than the selected MOFs will allow the diffusion of siloxane molecules with larger molecular weights, accounting for potential variations in mixture composition in practical applications. Additionally, MOFs labeled as not synthesizable in the QMOF data set were excluded. The QMOF database has 79 structures with this combination of properties. Given that only a small fraction of reported MOFs have been synthesized multiple times,^51^ we used the number of citations the original report of a MOF’s synthesis has received as a simple proxy for the ease of use of a MOF.^24,52^ Citation data for the original reports for each of the 79 materials were collected from Google Scholar up to April 2024. There are only 17 MOFs with more than 300 citations. In this work, we selected materials from this set of 17 MOFs. Citation data and pore parameters for these structures are listed in Table S1.

### 2.2 Computational Model

For molecular simulations, we applied our recently developed siloxane force field^23^ for each molecule and the Universal Force Field^53^ (UFF) for all MOF framework atoms. Dispersion interactions between the siloxanes and the MOF atoms were described using Lennard-Jones (LJ) parameters derived via Lorentz–Berthelot mixing rules. Since our siloxane force field omits point charges on the siloxane atoms, Coulombic interactions were excluded in the siloxane/MOF interactions. All interactions were truncated at 12.8 Å with tail corrections applied. MOFs were considered rigid during these simulations, using the crystal structure reported in the QMOF data set without further modifications. Simulation cells for MOFs were expanded as necessary to ensure that the shortest perpendicular distance was at least 26 Å, meeting the minimum image convention relative to the cutoff distance.

### 2.3 Computational Methods

Adsorption loadings for linear siloxanes (L2, L3 and L4) were computed using configurational bias Monte Carlo (CBMC) simulations, while adsorption loadings for cyclic siloxanes (D4, D5, and D6) were computed using continuous fractional component Monte Carlo (CFCMC) simulations. These simulations were performed with the RASPA simulation package.^54^ Molecular simulations were performed at *T* = 435 K, an operating temperature previously used in our study of the kinetic separation of linear and cyclic siloxanes in MOFs.^24^ Simulations were carried out using 10,000 cycles for equilibration and 400,000 cycles for production. Convergence tests that were used to establish these simulation parameters were sufficient are detailed in our previous work.^23^

The Ideal Adsorbed Solution Theory^55,56^ (IAST) was used to predict the multicomponent mixture adsorption using unary isotherms calculated from CBMC/CFCMC simulations as input. All GCMC-simulated unary isotherms were fitted using the dual-site Langmuir–Freundlich model. The dual-site Langmuir–Freundlich model was chosen because it captures the heterogeneous adsorption environments in MOFs, allowing accurate representation of both low loading and high loading adsorption regime. IAST calculations were performed with the RUPTURA simulation package.^57^

Molecular dynamics (MD) simulations were performed using LAMMPS.^58^ Self-diffusivity calculations were carried out at *T* = 435 K using a Nosé-Hoover thermostat.^59^ All MD simulations were carried out with a time step of 0.5 fs. Diffusivities were obtained from mean square displacements (MSD) that were computed using the order-*n* method.^60^ The initial configurations were generated from CBMC simulations using RASPA at *T* = 435 K and *p* = 1 atm. MD simulations were run in the canonical (*NVT*) ensemble. Three separate MD simulations were performed for each system. Each MD simulation comprised a 5 ns equilibration phase followed by at least 10 ns of production time to gather statistics.^61^

## 3 Results and Discussion

To gather initial data on the coadsorption of multicomponent siloxane mixtures in MOFs, we investigated a representative MOF with Refcode LUVTEC, which has a Helium void fraction of 0.43. LUVTEC was chosen because its moderate void fraction presents a balance between capacity and selectivity, making it a reasonable candidate for initial investigation. While high uptake capacity and high selectivity are desirable properties in separation processes, these two properties do not always go hand-in-hand.^62^ We first simulated single component isotherms of L2 and L3 and equimolar L2/L3 binary mixture isotherm in LUVTEC at 435 K. Figure 1a shows the comparison between CBMC simulated equimolar L2/L3 coadsorption in LUVTEC and IAST predictions. At fugacities below 10^6^ Pa, the bulk fluid mixture is in a vapor phase, as determined by the Peng–Robinson equation of state in RASPA. Selectivity at fugacities below 10^6^ Pa favors L3 adsorption over L2, which is the component with larger molecular weight and greater binding strength. Selectivity reversal occurs at fugacities above 10^6^ Pa, where the smaller species, L2, is preferentially adsorbed due to higher packing efficiency. This is an example of the entropy effect described above.

**Figure 1:**
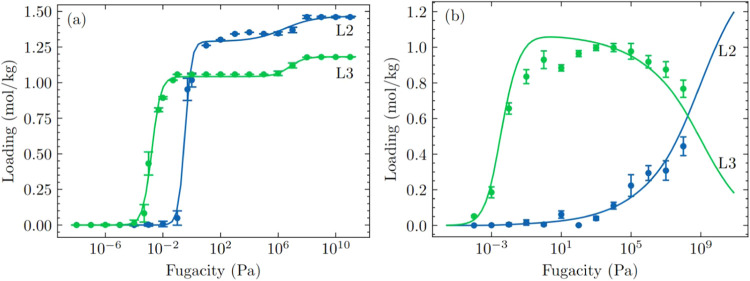
(a) Single component adsorption isotherms of L2 and L3 siloxanes in LUVTEC at 435 K. Production runs from CBMC simulations initialized with an amount of molecules equal to the number of adsorbed molecules at pore saturation for single component adsorption isotherms were averaged and are shown as dots, while solid lines represent the fitting to the dual-site Langmuir–Freundlich isotherm. (b) Equimolar binary mixture adsorption isotherm of L2 and L3 siloxanes in LUVTEC at 435 K. CBMC simulation data from four independent runs for the binary mixtures were averaged and are shown as dots, while solid lines represent the IAST predictions of the binary mixture.

Calculating multicomponent mixture adsorption isotherms for adsorption with liquid-like densities using molecular simulations is challenging due to low acceptance rates for particle insertions and deletions. At loadings approaching pore saturation (i.e., at fugacities above 10^5^ Pa in Figure 1b) larger uncertainties are observed in the CBMC simulation data. To ensure reliable results, CBMC simulations were performed with multiple independent runs to check for consistency. Two independent runs were conducted for single component adsorption isotherms of L2 and L3, with one initialized without any adsorbed molecules and the other with an amount of molecules equal to the number of adsorbed molecules at pore saturation. Four independent runs were performed for binary mixture adsorption isotherms, initialized without any molecules, saturated with L2, saturated with L3, and an equal number of L2 and L3 molecules. Figure S1 shows the CBMC simulation results from four independent runs for the equimolar binary L2/L3 mixture in LUVTEC at 435 K. The differences between the results from each initialization method are minor at fugacities below 10^5^ Pa, and increase at fugacities above 10^5^ Pa. While the results from multiple runs show that the overall trends are consistent, the observed fluctuations and uncertainties highlight the challenges of simulating adsorption at liquid-like densities using CBMC.

IAST has been commonly used to predict mixture adsorption isotherms from single component adsorption isotherms. We validated the applicability of IAST by comparing its predictions with CBMC simulations. We also used IAST to determine the initialization method for CBMC simulations to calculate single component adsorption isotherms by comparing their IAST predicted mixture adsorption isotherms to CBMC simulation data. CBMC simulated single component adsorption isotherms of L2 and L3 in LUVTEC at 435 K were fitted to the dual-site Langmuir–Freundlich model, which was then used in IAST to predict mixture adsorption.

Figure 1a shows the CBMC simulated single component adsorption isotherms of L2 and L3 in LUVTEC at 435 K (circles), initialized with a loading equal to the number of adsorbed molecules at pore saturation, along with fitted dual-site Langmuir–Freundlich isotherms (solid lines). There are subtle differences between these single component isotherms and the single component isotherms obtained from CBMC simulations initialized with empty frameworks (see Figure S2). Figure 1b shows the CBMC simulated equimolar L2/L3 coadsorption isotherm in LUVTEC at 435 K (dots) and IAST predictions using the dual-site Langmuir–Freundlich parameters fitted to the CBMC simulated single component adsorption isotherms (solid lines). The IAST predicted isotherms and CBMC simulation results for the equimolar L2/L3 binary mixture in LUVTEC in Figure 1b agree reasonably well.

Figure S2 shows the IAST predictions that result from single component isotherms from an alternate set of CBMC simulations that were initialized with empty frameworks. It is clear that the agreement between these IAST predictions and the mixture CBMC data is relatively poor. Applying IAST under conditions where the pore is filled with molecules requires integration of the single component isotherms from low loadings to near-saturation loadings. The qualitative differences in the IAST predictions in Figures 1b and S2 occur because of the differences in the near-saturation regimes of the single component isotherms used in IAST. As noted above, this is the regime in which it is challenging to achieve complete convergence of CBMC simulations. The single component isotherms in Figure 1a show slightly higher saturation loadings than the isotherms in Figure S2. Furthermore, applying IAST with the isotherms from Figure 1a gives predictions in near quantitative agreement with mixture CBMC data, unlike IAST predictions based on the isotherms in Figure S2. We therefore concluded that the simulation protocol used in Figure 1a (i.e., starting each simulation with a saturated pore) gave more reliable single component results than the protocol used in Figure S2 (i.e., starting each simulation with an empty pore). This situation is similar to the conclusions drawn by Jamdade et al. for simulation of pore filling by water in MOFs.^52^ Therefore, subsequent CBMC simulations for single component adsorption isotherms of all siloxanes in all investigated MOFs were initialized with an amount of molecules equal to the number of adsorbed molecules at pore saturation. These saturated configurations were generated at a fugacity of 10^12^ Pa and a temperature of 435 K, conditions chosen to ensure that the pores were fully occupied by the siloxanes.

Motivated by these results, we studied the coadsorption of an equimolar six-component mixture of linear (L2, L3 and L4) and cyclic (D4, D5 and D6) siloxanes in LUVTEC using IAST. Figure 2a shows the CBMC simulated single component adsorption isotherms of L2, L3, L4, D4, D5 and D6 in LUVTEC at 435 K along with fitted dual-site Langmuir–Freundlich isotherms. Figure 2b shows the predicted adsorption of equimolar L2/L3/L4/D4/D5/D6 mixture at 435 K in LUVTEC using IAST. Selectivity of the multicomponent mixture at fugacities below 0.001 Pa favors D6 adsorption, which is the component with the largest molecular weight among the molecules in this mixture and therefore the greatest binding strength. Selectivity reversal occurs at pressures above 0.001 Pa, where linear siloxanes are preferentially adsorbed.

**Figure 2:**
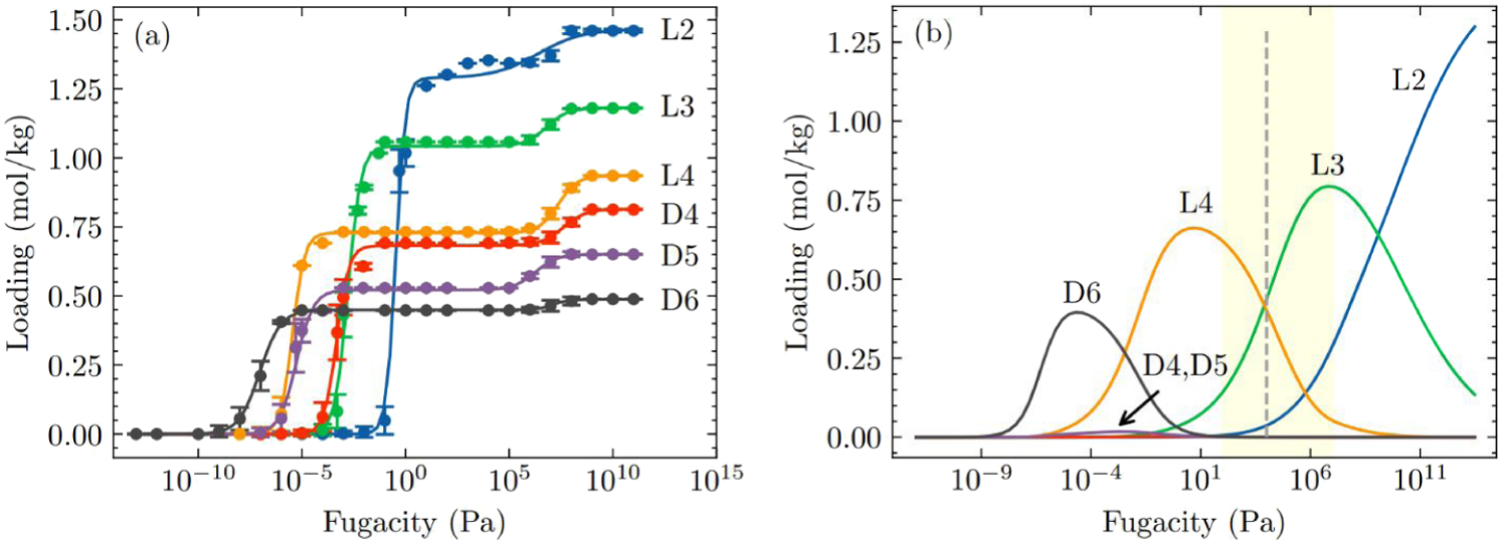
(a) Single component adsorption isotherms of L2, L3, L4, D4, D5 and D6 in LUVTEC at 435 K. CBMC simulation data are shown as dots, while solid lines represent the fitting to the dual-site Langmuir–Freundlich isotherm. (b) IAST estimated equimolar L2/L3/L4/D4/D5/D6 mixture adsorption in LUVTEC at 435 K. The shaded region indicates a typical pressure range for practical adsorption processes (100 Pa ≤ *P* ≤ 10^7^ Pa). The vertical dashed line indicates the pressure at which the bulk phase changes from vapor phase to a liquid phase, as determined by the Peng–Robinson equation of state in RASPA.^54^

At fugacities above 1 Pa, cyclic siloxanes are almost completely excluded from the pores and the adsorbed phase is almost exclusively a mixture of linear siloxanes. This observation is a straightforward consequence of the adsorption configurational entropy effect. At higher fugacities the adsorption of smaller linear siloxanes is progressively favored by entropic effects, until at the highest fugacities shown in Figure 2b the adsorbed phase is dominated by L2.

The results above for a specific MOF suggested that materials with larger void fractions and hence larger adsorption capacities would similarly exhibit selective adsorption of linear siloxanes relative to cyclic siloxanes due to configurational entropy effects. To test this idea we simulated single component isotherms of L2, L3, L4, D4, D5, and D6 siloxanes in FUNBOG, a MOF with a void fraction of 0.75, and used IAST to predict the six-component mixture adsorption. CBMC simulated single component adsorption isotherms of L2, L3, L4, D4, D5 and D6 in FUNBOG at 435 K along with fitted dual-site Langmuir–Freundlich isotherms are shown in Figure S3. Figure 3 shows that at fugacities above 1 Pa, linear siloxanes are selectively adsorbed in FUNBOG while cyclic siloxanes are excluded from the pores, following the same trend observed in LUVTEC.

**Figure 3:**
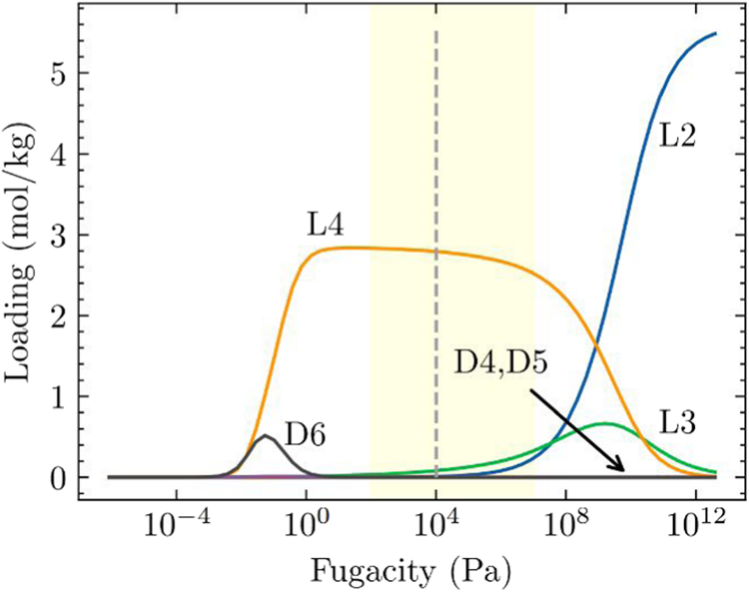
IAST estimated equimolar L2/L3/L4/D4/D5/D6 mixture adsorption in FUNBOG at 435 K. The shaded region indicates the typical pressure range for practical adsorption processes (100 Pa ≤ *P* ≤ 10^7^ Pa). The vertical dashed line indicates the pressure at which the bulk phase changes from vapor phase to a liquid phase, as determined by the Peng–Robinson equation of state in RASPA.^54^

Given the consistent findings that entropy effects drive the selective adsorption of linear siloxanes relative to cyclic siloxanes, extensive computational screening of MOFs was deemed unnecessary. Instead, we focused on selecting a MOF from the list of 17 potential candidates in Table S1 that would balance practical and performance considerations effectively. The selection of these 17 materials was described above. ZIF-70 emerged as an attractive candidate for several reasons. High uptake capacities are desirable in industrial applications for achieving longer breakthrough times and reducing the frequency of bed regeneration.^62^ The Helium void fraction of ZIF-70 is 0.62, while not the highest among the 17 materials (which range from 0.35 to 0.76), offers a substantial capacity for adsorption. The original report of ZIF-70 has received the highest number of citations among the materials we considered,^63^ a simple metric hinting at ease of synthesis. ZIF-70 was also reported to have high structural, thermal and chemical stability.^63–65^ These are important factors for scaling up from laboratory research to industrial applications. These factors combine to make ZIF-70 an attractive candidate material for further study, leading us to perform computational simulations to investigate its potential for the selective adsorption of linear and cyclic siloxanes.

Figure 4 shows IAST predicted mixture adsorption isotherms for an equimolar mixture of six component linear and cyclic siloxanes in ZIF-70. The results show the selective adsorption of linear siloxanes at pressures above ∼100 Pa due to configurational entropy effects. This selective adsorption behavior is consistent with our earlier findings and highlights the potential of ZIF-70 for effective separation processes based on adsorption equilibrium.

**Figure 4:**
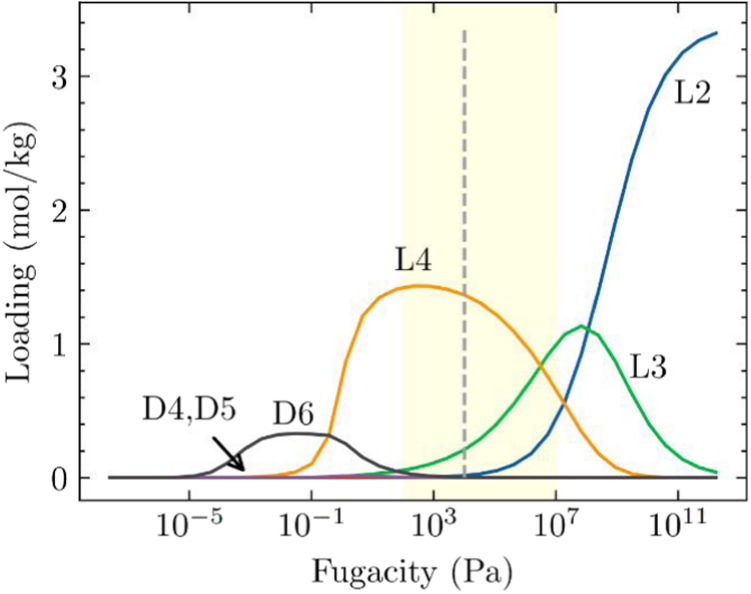
IAST estimated equimolar L2/L3/L4/D4/D5/D6 mixture adsorption in ZIF-70 at 435 K. The shaded region indicates the typical pressure range for practical adsorption processes (100 Pa ≤ *P* ≤ 10^7^ Pa). The vertical dashed line indicates the pressure at which the bulk phase changes from vapor phase to a liquid phase, as determined by the Peng–Robinson equation of state in RASPA.^54^

The analysis above using adsorption isotherms is of course only applicable if the adsorbent can reach equilibrium on reasonable time scales. We performed equilibrium MD simulations to calculate the self-diffusivities of the pure component L2, L3, L4, D4, D5, and D6 siloxanes in rigid ZIF-70 at 435 K to assess whether it is reasonable to expect that equilibrium can be reached rapidly for this MOF. To the best of our knowledge, no simulated or measured self-diffusivities of linear and cyclic siloxanes in the bulk state are available in the literature. However, experimental bulk viscosities of various linear and cyclic siloxanes at 298 K were reported by Dodgson et al.^66^ Using these viscosity data, we applied the Stokes–Einstein equation to estimate the self-diffusivities of siloxanes at 298 K. The procedure for calculating the bulk self-diffusivities of siloxanes is provided in the Supporting Information.

Figure 5 shows the self-diffusivities of the pure component L2, L3, L4, D4, D5, and D6 siloxanes at pore saturation concentrations in rigid ZIF-70 at 435 K (green dots), along with the estimated bulk siloxane self-diffusivities at 298 K (orange squares). The number of L2, L3, L4, D4, D5 and D6 siloxane molecules in these simulations was 66, 45, 34, 32, 25, and 23 respectively. The self-diffusivities generally decrease as the molecular weight of the siloxane molecule increases. Cyclic D4 exhibits a lower self-diffusivity than its linear counterpart, L4, which is consistent with observations in bulk liquid L4 and D4 siloxanes. In a separate set of MD simulations, we calculated the self-diffusivities of L2, L3, L4, D4, D5, and D6 in rigid ZIF-70 at 435 K, where each simulation contains 20 molecules in the framework. In our MD calculations at pore saturation concentrations in rigid ZIF-70 at 435 K, the self-diffusivity of D5 siloxane is higher than the self-diffusivities of L3, L4, and D4 siloxanes. For D6, its self-diffusivity is lower than that of D5, but higher than D4, and comparable to L3 and L4 siloxanes. Compared to the self-diffusivities calculated with an equal number of molecules for each siloxane species (20 molecules), the self-diffusivities at pore saturation are lower. This trend is consistent with observations in loading-dependent hydrocarbon diffusivities in porous materials, where self-diffusivities typically decrease as loading increases.^67,68^

**Figure 5:**
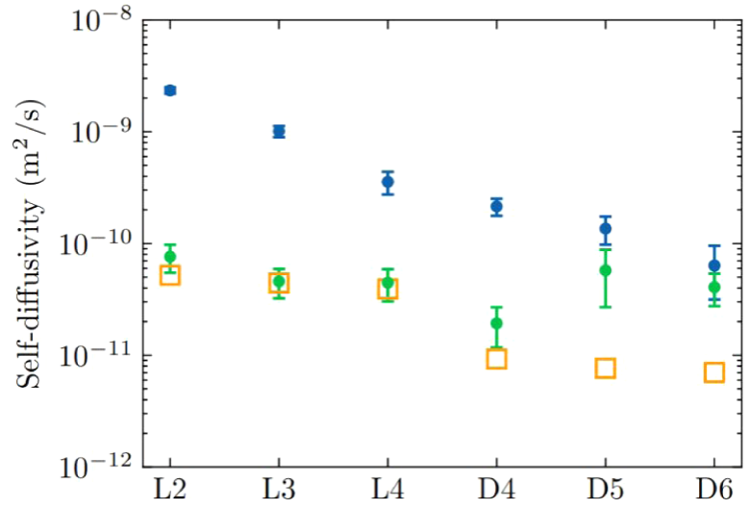
Self-diffusivities of L2, L3, L4, D4, D5, and D6 siloxanes at 435 K in rigid ZIF-70 compared to bulk self-diffusivities at 298 K. Green dots represent the self-diffusivities of siloxanes at pore saturation concentrations in rigid ZIF-70, while blue dots correspond to the self-diffusivities in rigid ZIF-70 with 20 molecules of each siloxane species. The orange squares show the bulk self-diffusivities estimated using the Stokes–Einstein equation from experimental viscosity data at 298 K.^66^

It is important to note that the above calculations were performed using a rigid ZIF-70 framework. Yang et al. showed that the diffusivities of flexible molecules in rigid MOFs are in many cases approximately 1 order of magnitude higher than in flexible MOFs.^69^ This observation suggests that if we were to account for framework flexibility the self-diffusivities calculated using a flexible ZIF-70 framework could be lower by an order of magnitude. However, even with this reduction, the diffusivities would be comparable to diffusivities of the molecules in their bulk liquid states and remain within the range of practical significance (10^–14^ to 10^–7^ m^2^/s),^69^ suggesting that adsorption equilibrium in ZIF-70 can be reached on reasonable time scales.

While the results presented so far have focused on equimolar six component mixture of linear and cyclic siloxanes, the permeate mixture a membrane separation system is unlikely to be an equimolar mixture, in part because molecular weight distribution of the mixture may not have a sharp cutoff. We used IAST to explore the implications of other mixture compositions in ZIF-70 at 435 K by varying the composition and number of components of the siloxane mixtures.

We first considered a nonequimolar six-component mixture of linear (L2, L3, L4) and cyclic (D4, D5, D6) siloxanes where the mole fractions of siloxanes in the bulk mixture are L2/L3/L4/D4/D5/D6 = 0.1/0.1/0.1/0.3/0.25/0.15. Figure 6a shows the IAST predicted adsorption isotherms in ZIF-70 at 435 K for this mixture. The results show that entropy effects continue to favor the selective adsorption of linear siloxanes over cyclic siloxanes in the pressure range relevant to practical adsorption processes. The adsorption behavior of this nonequimolar mixture is similar to that of an equimolar mixture, with entropy effects favoring the selective adsorption of linear siloxanes over cyclic siloxanes in the pressure range relevant to practical adsorption processes.

**Figure 6:**
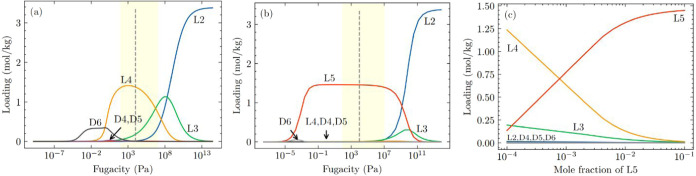
(a) IAST predicted adsorption isotherms for a nonequimolar six-component mixture of linear (L2, L3, L4) and cyclic (D4, D5, D6) siloxanes in ZIF-70 at 435 K. The mole fractions of the bulk mixture are L2/L3/L4/D4/D5/D6 = 0.1/0.1/0.1/0.3/0.25/0.15. The shaded region indicates a typical pressure range for practical adsorption processes (100 Pa ≤ *P* ≤ 10^7^ Pa). The vertical dashed line indicates the pressure at which the bulk phase changes from vapor phase to a liquid phase, as determined by the Peng–Robinson equation of state in RASPA.^54^ (b) IAST predicted adsorption isotherms for an equimolar seven-component mixture of linear (L2, L3, L4, L5) and cyclic (D4, D5, D6) siloxanes in ZIF-70 at 435 K. The shaded region indicates a typical pressure range for practical adsorption processes (100 Pa ≤ *P* ≤ 10^7^ Pa). The vertical dashed line indicates the pressure at which the bulk phase changes from vapor phase to a liquid phase, as determined by the Peng–Robinson equation of state in RASPA.^54^ (c) IAST predicted adsorbed loading seven-component mixture of linear (L2, L3, L4, L5) and cyclic (D4, D5, D6) siloxanes in ZIF-70 at 435 K as a function of mole fraction of L5 where the rest of the siloxanes (L2, L3, L4, D4, D5, and D6) are kept equimolar in the bulk mixture with the total fugacity fixed at 10^4^ Pa.

In another scenario, we considered an equimolar seven-component mixture linear (L2, L3, L4, L5) and cyclic (D4, D5, D6) siloxanes. Figure 6b shows the IAST predicted adsorption isotherms for this seven-component mixtures in ZIF-70 at 435 K. The results show that at practical adsorption pressures, entropy effects favor the selective adsorption of L5 over the cyclic siloxanes. The stronger binding strength of L5 relative to the rest of the linear siloxanes (L2, L3, and L4) leads to its preferential adsorption over L2, L3, and L4. Entropy effects that favor the adsorption of L2 and L3 over L4 and L5 are only observed at much higher fugacities, above 10^7^ Pa.

To understand the effect of L5 as a minor component in this seven-component mixture on the adsorbed phase in ZIF-70, we fixed the total fugacity at 10^4^ Pa and varied the mole fraction of L5 in the bulk mixture while keeping the other six components (L2, L3, L4, D4, D5, D6) equimolar. Figure 6c shows the effect of the concentration of L5 (expressed in mole fraction of L5 in this seven-component mixture) on the IAST predicted adsorbed loading in ZIF-70. At 0.01% mole fraction of L5 in this mixture, the adsorbed species is predominantly L4, followed by a smaller fraction of L3 and L5 in the adsorbed phase. As the concentration of L5 in the mixture increases, the amount of L5 adsorbed in ZIF-70 also increases, while the adsorption of L4 and L3 decreases. When the mole fraction of L5 exceeds 0.1%, L5 becomes the dominant adsorbed species, surpassing L4 and L3. At 10% mole fraction of L5 in this mixture, the adsorption of the other siloxanes (L2, L3, L4, D4, D5, D6) is almost completely excluded from the pores, and L5 is preferentially adsorbed.

The results above provide compelling evidence that several large-pore MOFs can achieve equilibrium separations of linear and branched siloxanes at a fixed temperature (435 K). The large void spaces in these MOFs are able to accommodate many siloxane molecules. The ability to achieve equilibrium quickly ensures that selectivity will not be kinetically limited but reflective of the preference for linear over cyclic siloxanes due to entropy effects. To develop a practical separation process, however, it is also important to be able to readily recover the adsorbed species. To consider recovery of adsorbed siloxanes from ZIF-70, we first explored a desorption process using a vacuum-temperature swing adsorption (VTSA) operation.

Thermogravimetric analysis (TGA) of ZIF-70 indicated that its decomposition temperature is 663 K.^63^ Based on this, we assumed a desorption temperature of 573 K is reasonable to examine the desorption of linear siloxanes. Because our calculations above showed that adsorption at 435 K could lead to situations where only linear siloxanes are adsorbed, we focused on the properties of linear siloxanes in ZIF-70 at 573 K. CBMC simulations were used to calculate single-component isotherms for linear siloxanes in ZIF-70 at 573 K and fitted to the dual-site Langmuir–Freundlich model (see Figure 7a). IAST was used to predict the equimolar L2/L3/L4 mixture adsorption isotherm in ZIF-70 at 573 K. We then defined the desorption pressure as the pressure at which the total adsorbed loading of linear siloxanes in ZIF-70 at 573 K is reduced to 25% of the total adsorbed loading of linear siloxanes at the adsorption conditions. The total adsorbed loading for linear siloxanes under the adsorption conditions is marked by the red star in Figure 7b. With this definition, the desorption pressure was determined to be 170 Pa (0.0017 bar), indicated by the purple triangle in Figure 7b. This is a deep level of vacuum, which may cause challenges in practical settings, but suggests that VTSA is potentially worth consideration for the separation of linear and cyclic siloxanes.

**Figure 7:**
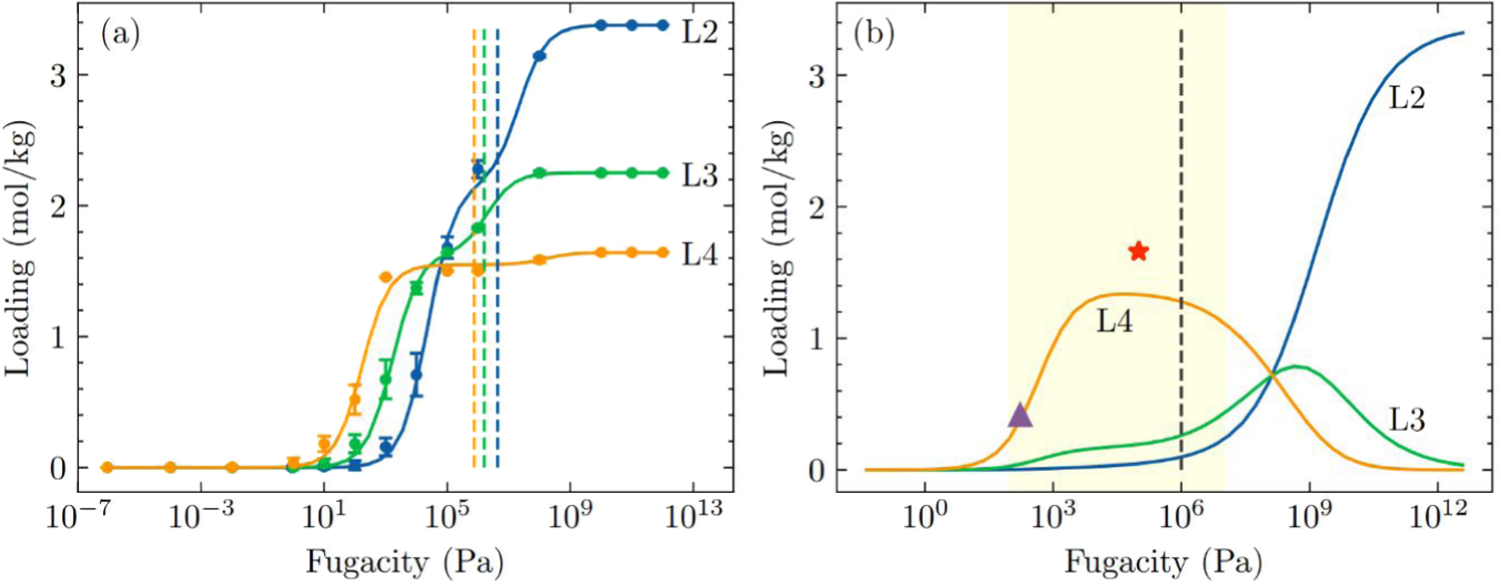
(a) Single component adsorption isotherms of L2, L3 and L4 in ZIF-70 at 573 K. CBMC simulation data are shown as dots, while solid lines represent the fitting to the dual-site Langmuir–Freundlich isotherm. Vertical dashed lines indicate the vapor pressures of the linear siloxanes at 573 K from the NIST Webbook.^70^ (b) IAST estimated equimolar L2/L3/L4 mixture adsorption in ZIF-70 at 573 K. The shaded region indicates a typical pressure range for practical adsorption processes (100 Pa ≤ *P* ≤ 10^7^ Pa). The vertical dashed line indicates the pressure at which the bulk phase changes from vapor phase to a liquid phase as determined by the Peng–Robinson equation of state in RASPA.^54^ The red star represents the total adsorbed linear siloxanes at a pressure and temperature of 10^5^ Pa and 435 K, while the purple triangle represents the total adsorbed linear siloxanes under the desorption pressure and temperature, 170 Pa and 573 K.

Given the deep vacuum that would be required with VTSA to desorb 25% of the adsorbed linear siloxanes, we explored an alternative desorption strategy using supercritical CO_2_. Supercritical CO_2_ is commonly used to activate MOFs.^71–75^ Various reports have described the use of supercritical CO_2_ for the regeneration of activated carbon adsorbed with sulfur compounds,^76^ toluene,^77^ and other organic compounds^78^ due to its high diffusivity in micropores and low surface tension.^79^ CO_2_ is a small molecule compared to linear siloxanes, so we hypothesized that a sufficiently high partial pressure of CO_2_ in a mixture with linear siloxanes would drive the selective adsorption of CO_2_ due to entropy effects, thereby desorbing the linear siloxanes.

To test this hypothesis, we used IAST to predict CO_2_/L2/L3/L4 mixture coadsorption with low bulk phase composition of linear siloxanes in ZIF-70 at 435 K. At each pressure state point the partial pressure of each linear siloxane was set to 0.1% of their vapor pressure at 435 K. The vapor pressures of L2, L3, and L4 at 435 K are listed in Table S5. Figure 8 shows the IAST predicted adsorbed loading in ZIF-70. At a total fugacity of 200 bar, the adsorbed loading of linear siloxanes in ZIF-70 is reduced by 25%, as indicated by the black triangle in Figure 8. At total fugacities above 200 bar, CO_2_ is preferentially adsorbed, and linear siloxanes are virtually excluded from the pores. This indicates that desorption of linear siloxanes can be achieved by the preferential adsorption of CO_2_ at sufficiently high pressures due to entropy effects.

**Figure 8:**
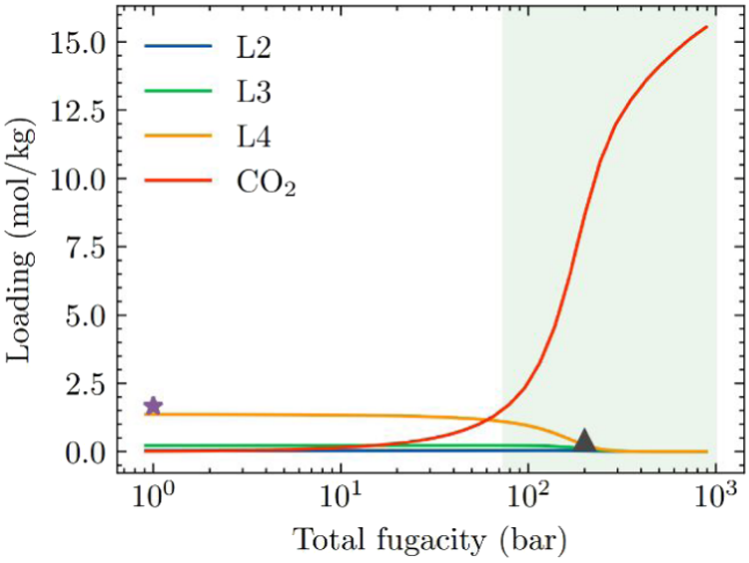
IAST estimated L2/L3/L4/CO_2_ mixture adsorption in ZIF-70 at 435 K. At each pressure state point the partial pressure of each linear siloxane was set to 0.1% of their vapor pressure at 435 K. The purple star represents the total adsorbed linear siloxanes at a pressure of 1 bar, while the black triangle represents the total adsorbed linear siloxanes under the desorption pressure of 200 bar. The region shaded in green indicates the pressure range at which CO_2_ is a supercritical fluid at this temperature.

The results presented in this study demonstrate that configurational entropy effects drive the selective adsorption of linear siloxanes over cyclic siloxanes in MOFs. Both LUVTEC and FUNBOG show that at sufficiently high pressures, linear siloxanes are preferentially adsorbed due to their higher packing efficiency. ZIF-70 emerged as a potential candidate for practical applications due to its ease of synthesis and high stability. The feasibility of using a VTSA process for the equilibrium separation of linear and cyclic siloxanes was demonstrated, which resulted in a 75% reduction in adsorbed phase concentration of linear siloxanes. The potential for supercritical CO_2_ to drive the desorption of linear siloxanes as an alternative strategy for the regeneration of ZIF-70 was also considered.

## 4 Summary

We presented computational results to evaluate the potential of MOFs for the equilibrium separation of linear and cyclic siloxanes. Our calculations used force fields that were shown in earlier work to quantitatively describe the bulk phase behavior of siloxanes and the adsorbed phase of siloxanes in MOFs.^23^ We used a combination of CBMC simulations and IAST to predict the adsorption behavior of equimolar six-component mixture of linear and cyclic siloxanes in a selection of synthesizable MOFs with medium to large pore volumes. Test calculations using a prototypical MOF showed that IAST gave accurate predictions of multicomponent adsorption. Configurational entropy effects drive the selective adsorption of linear siloxanes and virtually excludes the adsorption of cyclic siloxanes in the typical pressure range for practical adsorption processes.

Using the number of citations as a proxy for synthesizability of a MOF, we identified ZIF-70 as a promising adsorbent for the equilibrium separation of linear and cyclic siloxanes. Equilibrium MD simulations were performed to calculate the self-diffusivities of the siloxanes in rigid ZIF-70, showing that adsorption equilibrium can be achieved on reasonable time scales. We extended our analysis to nonequimolar mixtures and variations in molecular weight distribution, confirming the preferential adsorption of linear siloxanes over cyclic siloxanes due to configurational entropy effects. Additionally, we explored the feasibility of using VTSA for the regeneration of ZIF-70 after adsorption of linear siloxanes. We also investigated the use of supercritical CO_2_ as an alternative strategy for sorbent regeneration and recovery of adsorbed linear siloxanes. IAST predictions showed that at pressures above 200 bar and 435 K, preferential adsorption of CO_2_ due to entropy effects displaced the adsorbed linear siloxanes in ZIF-70.

Overall, our results indicate that MOFs have potential as adsorbents for equilibrium separation of cyclic and linear siloxanes. ZIF-70 emerged as a promising candidate for further experimental testing. It is important to note that our simulations were performed on rigid, defect-free MOF frameworks and we only considered MOFs that do not have open metal sites. In practical applications, structural defects such as missing linkers,^80,81^ could potentially create open metal sites that might catalyze reactions of siloxanes. Since the adsorption of siloxanes reaches liquid-like densities, framework flexibility effects could impact equilibrium capacity. Prior studies showed that structural flexibility of ZIFs affects the diffusion selectivity of gas mixture and could undergo structural changes during liquid-phase adsorption.^82–85^ The absolute loadings of siloxanes may be marginally underestimated in the rigid framework, however the preferential adsorption of linear siloxanes driven by entropy effects remains valid. It may be useful in the future to assess and quantify the possible impact of pore swelling on the adsorption of siloxanes by incorporating framework flexibility effects.^86,87^

## Supplementary Material





## Notes

Additional data and computational results: citation data and pore parameters for the 17 MOFs, single component adsorption isotherms in FUNBOG and ZIF-70, procedure to estimate the self-diffusivities of bulk liquid linear and cyclic siloxanes from experimental viscosity data. Complete collection of data for unary and binary adsorption isotherms of L2 and L3 in LUVTEC from CBMC simulations and IAST predictions; single component adsorption isotherms data of L2, L3, L4, D4, D5 and D6 in LUVTEC, FUNBOG and ZIF-70 from CFCMC and CBMC simulations; multicomponent adsorption isotherms data of L2, L3, L4, L5, D4, D5, D6 in LUVTEC, FUNBOG and ZIF-70 from IAST predictions. Figures illustrating the siloxane molecules of interests and the MOFs ZIF-70, LUVTEC, and FUNBOG (PDF)
Simulation data (XLSX)
