# Supplementary material for: Equilibrium Separation of Siloxanes in Metal–Organic Frameworks
Source: J Phys Chem C Nanomater Interfaces. 2025 Sep 4;129(37):16844–54. doi: 10.1021/acs.jpcc.5c04283 (PMC12451738; doi:10.1021/acs.jpcc.5c04283)
Supplement: Supplementary file 1 [file jp5c04283_si_001.pdf]

Supporting Information: Equilibrium Separation of Siloxanes in Metal-Organic Frameworks

Jia Yuan Chng† and David S. Sholl*,‡

†School of Chemical & Biomolecular Engineering, Georgia Institute of Technology, Atlanta, Georgia 30332-0100, United States.

‡Oak Ridge National Laboratory, Oak Ridge, Tennessee 37830, United States.

E-mail: shollds@ornl.gov

All simulation data are collated in a single Excel spreadsheet that is available within the publication's Supporting Information.

Sheet name	Description
ZIF70_L2_T435	unary isotherm of L2 in ZIF-70 at 435 K, fitted isotherm parameters
ZIF70_L3_T435	unary isotherm of L3 in ZIF-70 at 435 K, fitted isotherm parameters
ZIF70_L4_T435	unary isotherm of L4 in ZIF-70 at 435 K, fitted isotherm parameters
ZIF70_L5_T435	unary isotherm of L5 in ZIF-70 at 435 K, fitted isotherm parameters
ZIF70_D4_T435	unary isotherm of D4 in ZIF-70 at 435 K, fitted isotherm parameters
ZIF70_D5_T435	unary isotherm of D5 in ZIF-70 at 435 K, fitted isotherm parameters
ZIF70_D6_T435	unary isotherm of D6 in ZIF-70 at 435 K, fitted isotherm parameters
ZIF70_CO2_T435	unary isotherm of CO2 in ZIF-70 at 435 K, fitted isotherm parameters
ZIF70_L2_T573	unary isotherm of L2 in ZIF-70 at 573 K, fitted isotherm parameters
ZIF70_L3_T573	unary isotherm of L3 in ZIF-70 at 573 K, fitted isotherm parameters
ZIF70_L4_T573	unary isotherm of L4 in ZIF-70 at 573 K, fitted isotherm parameters
FUNBOG_L2_T435	unary isotherm of L2 in FUNBOG at 435 K, fitted isotherm parameters
FUNBOG_L3_T435	unary isotherm of L3 in FUNBOG at 435 K, fitted isotherm parameters
FUNBOG_L4_T435	unary isotherm of L4 in FUNBOG at 435 K, fitted isotherm parameters
FUNBOG_D4_T435	unary isotherm of D4 in FUNBOG at 435 K, fitted isotherm parameters
FUNBOG_D5_T435	unary isotherm of D5 in FUNBOG at 435 K, fitted isotherm parameters
FUNBOG_D6_T435	unary isotherm of D6 in FUNBOG at 435 K, fitted isotherm parameters
LUVTEC_L2_T435	unary isotherm of L2 in LUVTEC at 435 K initialized at pore saturation, fitted isotherm parameters
LUVTEC_L3_T435	unary isotherm of L3 in LUVTEC at 435 K initialized at pore saturation, fitted isotherm parameters
LUVTEC_L4_T435	unary isotherm of L4 in LUVTEC at 435 K initialized at pore saturation, fitted isotherm parameters
LUVTEC_D4_T435	unary isotherm of D4 in LUVTEC at 435 K initialized at pore saturation, fitted isotherm parameters
LUVTEC_D5_T435	unary isotherm of D5 in LUVTEC at 435 K initialized at pore saturation, fitted isotherm parameters
LUVTEC_D6_T435	unary isotherm of D6 in LUVTEC at 435 K initialized at pore saturation, fitted isotherm parameters
LUVTEC_L2_T435_nonsat	unary isotherm of L2 in LUVTEC at 435 K initialized w/o molecules, fitted isotherm parameters
LUVTEC_L3_T435_nonsat	unary isotherm of L3 in LUVTEC at 435 K initialized w/o molecules, fitted isotherm parameters
LUVTEC_L2L3_T435	binary mixture isotherms of L2 and L3 in LUVTEC at 435 K
selfDiffusivity	self-diffusivities of siloxanes in ZIF-70 at 435 K calculated from MD simulations

Table S1: Citation data from Google scholar up to April 2024 and pore parameters of the 17 MOFs from the QMOF database.[1]

QMOF ID	RefCode	Common name	PLD (A)	LCD (A)	Void fraction	# citations
qmof-2d8d50c	COJHIT	-	9.3	10.1	0.63	389
qmof-da6fcce	VEBHUG	IRMOF-20	9.4	17.4	0.82	1667
qmof-66acad7	QUQBEL	-	9.4	9.8	0.62	796
qmof-3fb0086	IBICAZ	-	9.7	9.9	0.38	349
qmof-5c354ae	JOYKUF	-	9.8	9.9	0.35	1010
qmof-e8046f4	IBICED	-	9.9	10.0	0.37	349
qmof-aaa921d	LUVTEC	-	10.0	10.5	0.43	602
qmof-8b6046e	JIZJAF	-	10.0	10.5	0.4	379
qmof-b1cbe7a	QUPZIM02	-	10.2	10.4	0.65	743
qmof-9d2a0e4	FUNBOG	-	10.6	12.8	0.75	327
qmof-b46c098	COKNIB	-	10.7	11.5	0.59	386
qmof-07cc468	JOYKOZ	-	11.3	11.5	0.43	922
qmof-c80cf5d	LIHFAK	-	11.9	14.7	0.76	612
qmof-b927353	FUNCEX	-	12.5	13.0	0.76	327
qmof-1c956df	GITVEL01	ZIF-70	13.3	15.1	0.62	4076
qmof-f7478bc	YUXQIS	-	14.3	17.4	0.68	582
qmof-b90158f	XIGFOJ	-	18.5	18.7	0.69	340

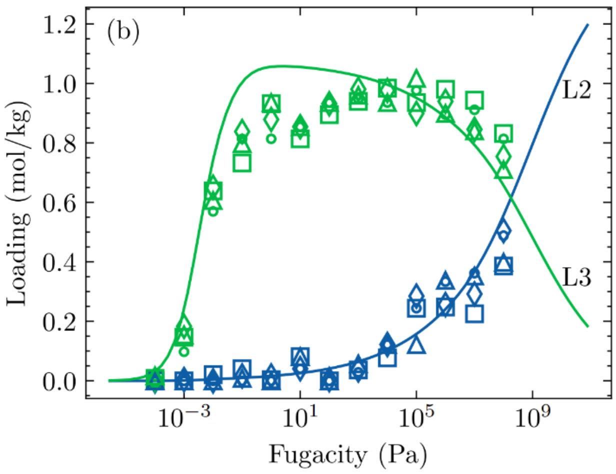

Figure S1: Equimolar binary mixture adsorption isotherm of L2 and L3 siloxanes in LUVTEC at 435 K. CBMC simulation data from four independent runs for the binary mixtures, initialized without any molecules, saturated with L2, saturated with L3, and equal number of L2 and L3 molecules, are shown as dots, squares, diamonds and triangles respectively. The solid lines represent the IAST predictions of the binary mixture using fitted dual-site Langmuir–Freundlich isotherm from CBMC simulations of single component isotherms initialized with loading equal to the number of adsorbed molecules at pore saturation.

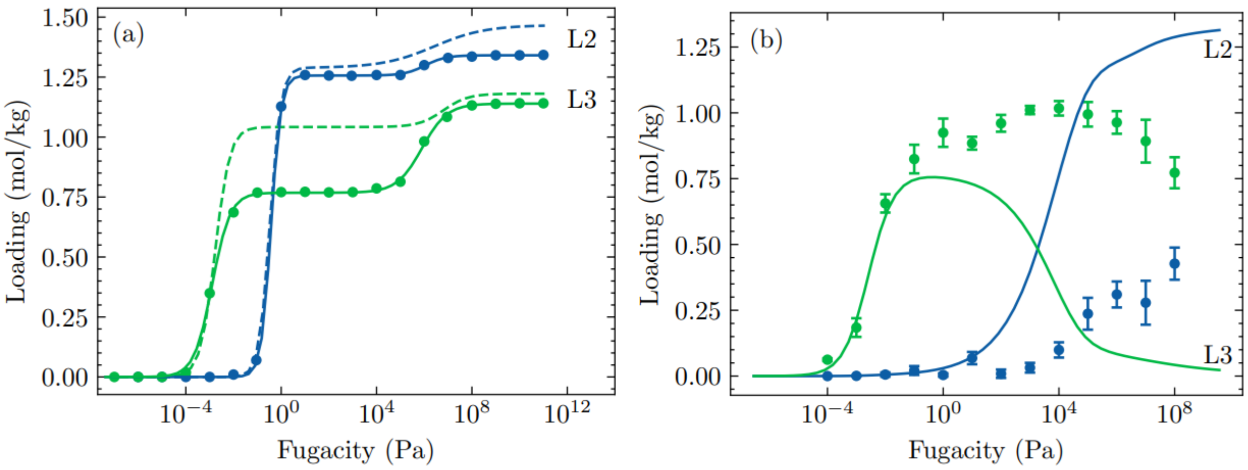

Figure S2: (a) Single component adsorption isotherms of L2 and L3 siloxanes in LUVTEC at 435 K. CBMC simulations initialized without any molecules for single component adsorption isotherms are shown as dots, while solid lines represent the fitting to the dual-site Langmuir–Freundlich isotherm. The dashed lines represent the fitting to the dual-site Langmuir–Freundlich isotherm from CBMC simulations initialized with loading equal to the number of adsorbed molecules at pore saturation (as shown in Figure 1a). (b) Equimolar binary mixture adsorption isotherm of L2 and L3 siloxanes in LUVTEC at 435 K. CBMC simulation data from four independent runs for the binary mixtures were averaged and are shown as dots, while solid lines represent the IAST predictions of the binary mixture.

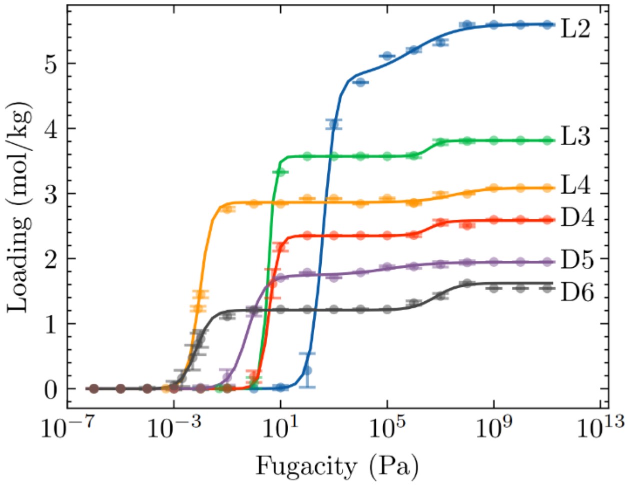

Figure S3: Single component adsorption isotherms of L2, L3, L4, D4, D5 and D6 siloxanes in FUNBOG at 435 K. CBMC simulation data are shown as dots, while solid lines represent the fitting to the dual-site Langmuir–Freundlich isotherm.

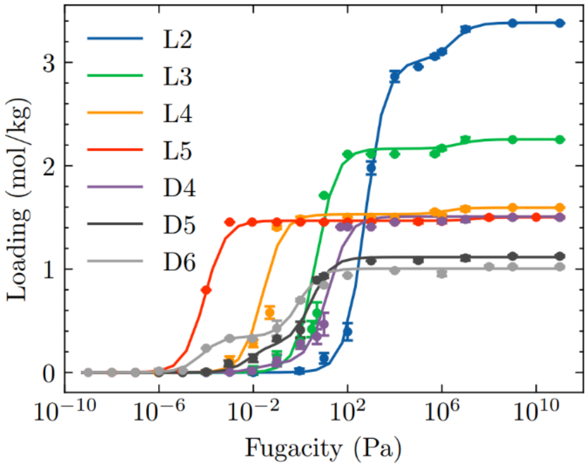

Figure S4: Single component adsorption isotherms of L2, L3, L4, L5, D4, D5 and D6 siloxanes in ZIF-70 at 435 K. CBMC simulation data are shown as dots, while solid lines represent the fitting to the dual-site Langmuir–Freundlich isotherm.

Estimation of self-diffusivities of linear and cyclic siloxanes using the Stokes-Einstein equation

Table S2 shows the experimental bulk viscosities η at 298 K of the cyclic (D4, D5, D6) and linear (L2, L3, L4) siloxanes reported by Dodgson et al.[2]

Self-diffusivities D of siloxanes can be estimated using the Stokes-Einstein equation for ellipsoidal molecules:[3]

$$D = \frac{k_B T}{6\pi\eta a(\ln(2a/b))^{-1}}$$

where k_B is the Boltzmann's constant, T is the temperature, η is the viscosity, a is the radius of the ellipsoid's major axis and b is the radius of the ellipsoid's minor axis. The ellipsoid radii for siloxanes are calculated using the minimum enclosing ellipsoid method (see Table S3).[4] The estimated bulk self-diffusivities at 298 K of the cyclic (D4, D5, D6) and linear (L2, L3, L4) are shown in Table S4.

Table S2: Experimental bulk viscosities at 298 K (η298) for cyclic (D4, D5, D6) and linear (L2, L3, L4) siloxanes.[2]

Siloxane	η_{298} (10^{-3} kg m^{-1} s^{-1})
D4	17.98
D5	21.70
D6	21.95
L2	3.91
L3	4.52
L4	5.03

Table S3: Ellipsoid diameters for siloxanes calculated using the minimum enclosing ellipsoid method.[4]

Siloxane	a (Å)	b (Å)
D4	11.28	9.56
D5	12.80	9.67
D6	13.85	10.43
L2	11.38	7.90
L3	15.36	7.90
L4	17.35	7.90

Table S4: Bulk self-diffusivities of cyclic (D4, D5, D6) and linear (L2, L3, L4) siloxanes at 298 K estimated using the Stokes-Einstein equation.

Siloxane	$\ln\left(\frac{2a}{b}\right)$	D_{298}(10^{-11} m^2/s)
D4	0.86	0.92
D5	0.97	0.77
D6	0.98	0.39
L2	1.06	5.19
L3	1.36	4.43
L4	1.48	3.91

Table S5: Vapor pressures of L2, L3 and L4 siloxanes at 435 K from the NIST Webbook.[5]

Siloxane	Vapor pressure (Pa)
L2	449928
L3	128630
L4	42524

Figures of MOFs and siloxanes

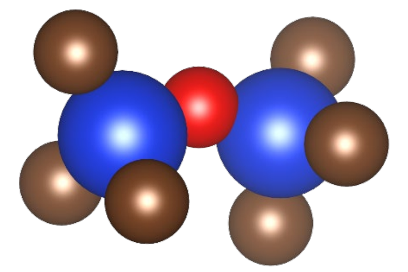	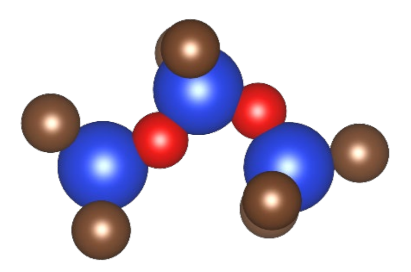	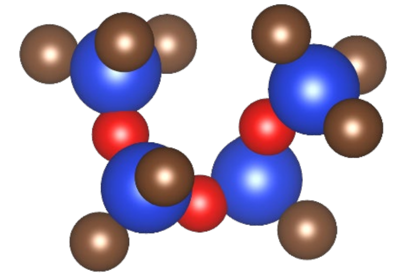
L2	L3	L4
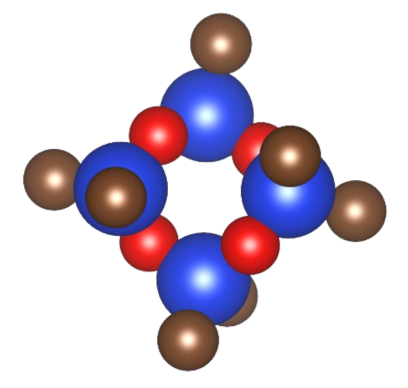	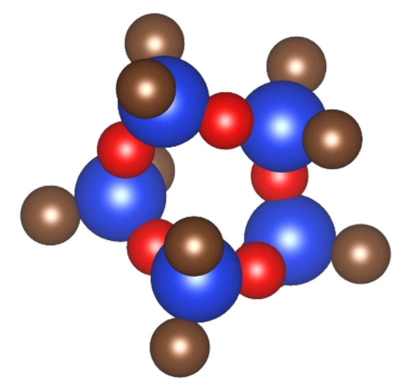	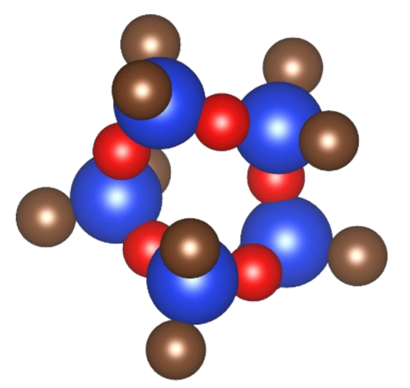
D4	D5	D6

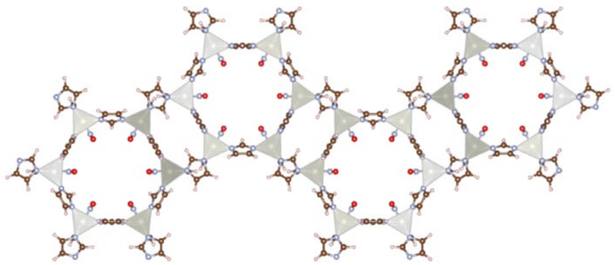

ZIF-70

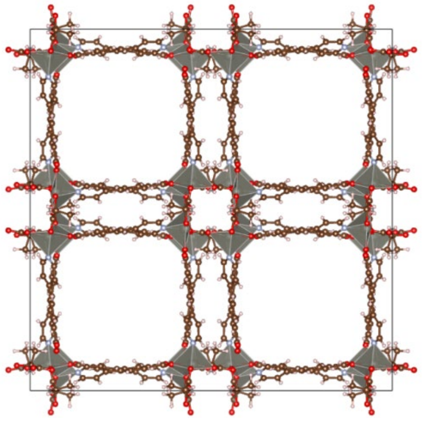

LUVTEC

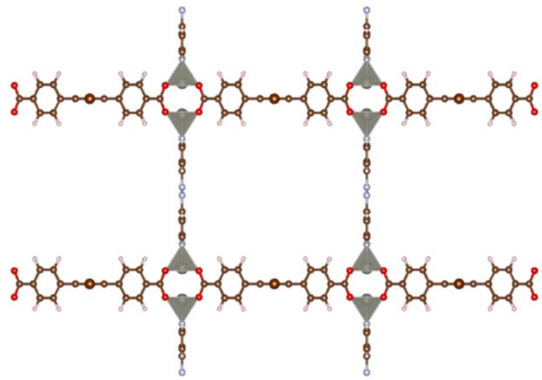

FUNBOG

References

1. Rosen, A. S.; Iyer, S. M.; Ray, D.; Yao, Z.; Aspuru-Guzik, A.; Gagliardi, L.; Notestein, J. M.; Snurr, R. Q. Machine Learning the Quantum-Chemical Properties of Metal–organic Frameworks for Accelerated Materials Discovery. Matter 2021, 4, 1578– 1597.
2. Dodgson, K.; Bannister, D.; Semlyen, J. Studies of Cyclic and Linear Poly(Dimethyl Siloxanes). 4. Bulk Viscosities. Polymer 1980, 21, 663–667.
3. Yang, K.; Lu, C.; Zhao, X.; Kawamura, R. From Bead To Rod: Comparison of Theories by Measuring Translational Drag Coefficients of Micron-Sized Magnetic Bead-Chains in Stokes Flow. PLOS ONE 2017, 12, 1–18.
4. Yu, X.; Tang, D.; Chng, J. Y.; Sholl, D. S. Efficient Exploration of Adsorption Space for Separations in Metal–Organic Frameworks Combining the Use of Molecular Simulations, Machine Learning, and Ideal Adsorbed Solution Theory. The Journal of Physical Chemistry C 2023, 127, 19229–19239.
5. Linstrom, P.; Mallard, W. NIST Chemistry WebBook, NIST Standard Reference Database Number 69. National Institute of Standards and Technology 2022.
